# AVDOS-VR: Affective Video Database with Physiological Signals and Continuous Ratings Collected Remotely in VR

**DOI:** 10.1038/s41597-024-02953-6

**Published:** 2024-01-25

**Authors:** Michal Gnacek, Luis Quintero, Ifigeneia Mavridou, Emili Balaguer-Ballester, Theodoros Kostoulas, Charles Nduka, Ellen Seiss

**Affiliations:** 1 Centre for Digital Entertainment, Faculty of Media and Communication, Bournemouth University, Poole, BH12 5BB UK; 2 Emteq Labs, Brighton, BN1 9RS UK; 3 Department of Computer and Systems Sciences, Stockholm University, 164 55 Stockholm, Sweden; 4 Department of Computing and Informatics, Faculty of Science and Technology, Interdisciplinary Neuroscience Research Centre, Bournemouth University, Poole, BH12 5BB UK; 5 Department of Information and Communication Systems Engineering, University of the Aegean, Karlovasi, 832 00 Greece; 6 Department of Psychology, Faculty of Science and Technology, Interdisciplinary Neuroscience Research Centre, Bournemouth University, Poole, BH12 5BB UK

**Keywords:** Human behaviour, Bioinformatics, Predictive markers, Scientific data, Databases

## Abstract

Investigating emotions relies on pre-validated stimuli to evaluate induced responses through subjective self-ratings and physiological changes. The creation of precise affect models necessitates extensive datasets. While datasets related to pictures, words, and sounds are abundant, those associated with videos are comparatively scarce. To overcome this challenge, we present the first virtual reality (VR) database with continuous self-ratings and physiological measures, including facial EMG. Videos were rated online using a head-mounted VR device (HMD) with attached emteqPRO mask and a cinema VR environment in remote home and laboratory settings with minimal setup requirements. This led to an affective video database with continuous valence and arousal self-rating measures and physiological responses (PPG, facial-EMG (7x), IMU). The AVDOS-VR database includes data from 37 participants who watched 30 randomly ordered videos (10 positive, neutral, and negative). Each 30-second video was assessed with two-minute relaxation between categories. Validation results suggest that remote data collection is ecologically valid, providing an effective strategy for future affective study designs. All data can be accessed via: www.gnacek.com/affective-video-database-online-study.

## Background & Summary

Conscious and subconscious affect recognition is a cornerstone of social interaction between humans and is one of the aspects of computer-human interaction we are yet to understand fully^1^. The continuous growth of affective computing (AC) research and literature is thriving towards objectively measuring and understanding affect and emotions in environmental contexts. Affective computing research communities are continuously exploring the design of affect-aware artificial systems^2^. The challenge remains in gaining insight into emotions, an inherently internal function to the outside observer^3^.

Affect detection models are typically generated through a multistage process, which consists of recording biological markers and subjective experiences simultaneously through ratings or questionnaires. Multiple sensors are often combined to form a more complete picture of affect through multi-modal classification^4^. The captured data is then used to model the relationship between these markers and subjective experiences to make predictions regarding the felt emotion^5^. This approach typically requires standardised stimulus databases and large datasets of affect measurements.

There is a sustained drive towards more reliable affective databases, designed to facilitate new insights by keeping up with changing technologies^6–8^. Videos are a relatively new addition to classic affective databases compared to other emotion induction methods, such as pictures, sounds, and words. Nevertheless, videos have become popular amongst researchers as a viable tool for eliciting emotional responses in experimental settings and VR^9,10^. Several affective video databases of varying sizes, lengths and measures have been developed such as LIRIS-ACCEDE^11^, VASD^12^, DEVO^13^ or CAAV^14^.

**Fig. 1:**
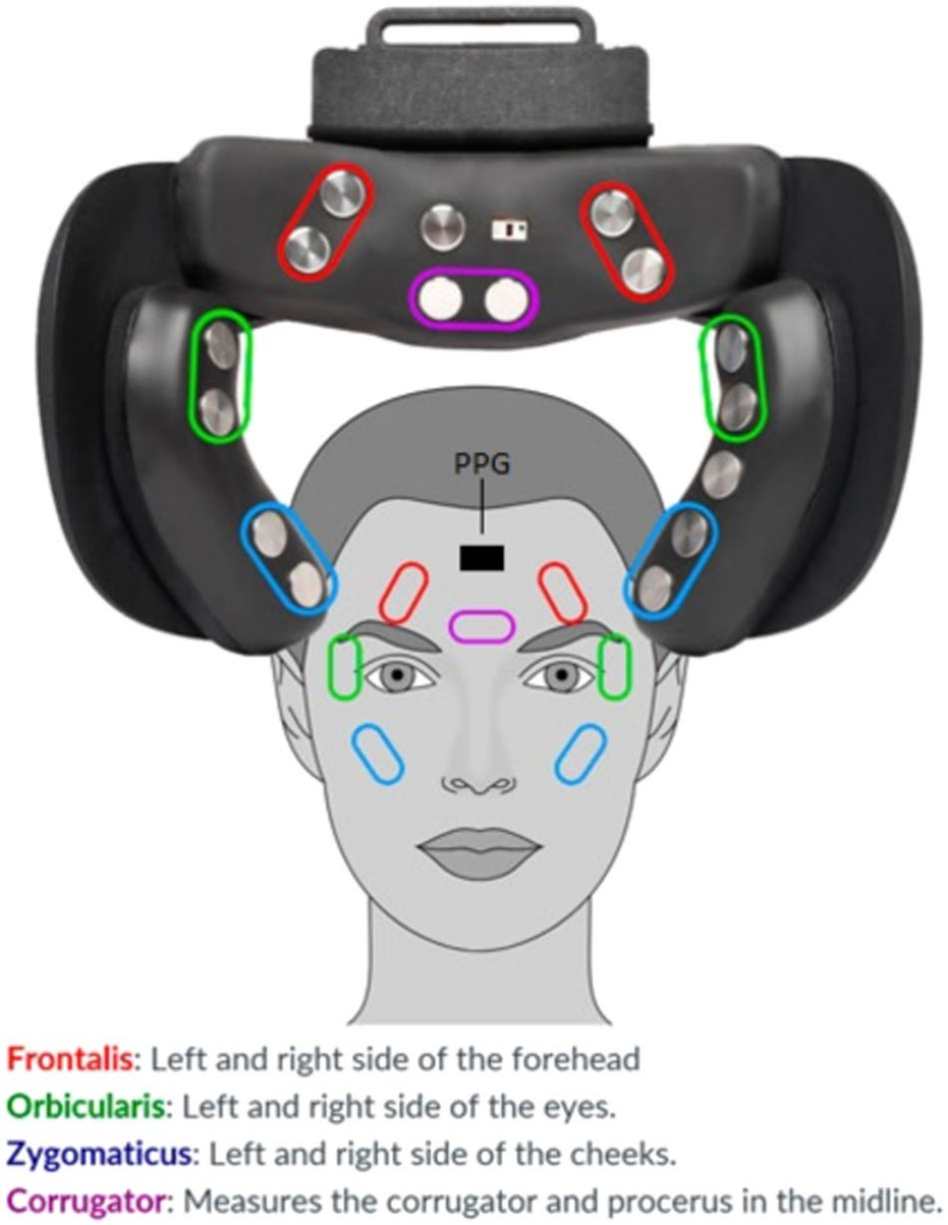
The mapping between seven EMG sensors on the emteqPRO and facial muscle groups. Note also the location of the forehead PPG sensor.

However, these have shortcomings. Video stimuli are complex structures with multiple variables, including frame rate, audio, duration, plot development, and camera angles. These factors greatly influence the emotional impact of videos^15^. Altering the duration of validated videos can compromise their intended emotional induction. Indeed, stimulus features such as duration, as well as visual and auditory properties, are crucial variables in databases equipped with self-ratings and physiological measures for several reasons. Firstly, studies on heart rate variability (HRV) recommend a minimum recording time of 30 seconds for reliable data^16^. Other physiological measures, such as heart rate, galvanic skin response (GSR), electromyography (EMG), and cortisol levels, exhibit varying response times^17^. Secondly, many databases rely on end-of-stimulus self-ratings, which may introduce biases and reduce accuracy, particularly with longer duration stimuli^18,19^. A solution for this is to collect continuous self-ratings for arousal and valence throughout the experience, as done by several studies^5,11^. Thirdly, a large proportion of studies investigating affect detection using physiological signals have been carried out in laboratory settings but with increasing availability, reliability and ease of use of wearable sensors^20^, more recent studies have been attempting to replicate the results outside the confines of heavily controlled laboratory environments^21^, e.g. in home settings. For this goal, we used the emteqPRO device - a VR HMD augmented with an array of sensors^22^ including seven channel EMG, a PPG and an inertial measurement unit (IMU) sensors (see Fig. 1). Previous studies robustly validated emteqPRO sensors for heart rate detection^23^, facial expressions^24^, breathing rate estimation^25^, valence and arousal^26,27^, and even pain perception^28^, enabling us to use this device to generate a novel, comprehensive database.

In summary, VR-based affective computing is groundbreaking since it has the potential to bridge controlled laboratory settings and real-world environments. VR, combined with dedicated sensors, offers an immersive platform to study emotions. This approach allows the fusion of perceptual and physiological data, facilitating a holistic understanding of emotions. It is a shift in research methodologies, providing valuable insights beyond traditional approaches^29^. The increasing popularity of VR as a research tool^30,31^ has resulted in more and more studies using videos, interactive VR content and in embedding physiological measures in VR paradigms^32^. However, there are only a few VR databases for affect detection that combine continuous self-ratings with a range of physiological measures^33,34^. These databases still lack key physiological measures for affect detection and, arguably, the most relevant physiological response underlying the valence dimension - facial micro-expressions^35,36^.

The need for a new video database for VR environments arises from the limitations of existing video-based datasets; fostering the shift towards VR-based studies featuring more immersive, easily controllable environments. Traditional datasets feature short video clips, which may not fully capture emotional dynamics. This problem is addressed with the AVDOS-VR database (***Affective Video Database Online Study - Virtual Reality***) presented in this paper.

The database adds to existing affective video databases with a novel approach by combining continuous self-ratings via a VR controller and multi-modal, physiological measures (Table 1) of standardised 30s-long videos presented via a VR HMD device. Longer VR videos offer more immersive and ecologically valid experiences, enhancing our understanding of emotions. Additionally, the unique array of facial sensors in VR, such as the emteqPRO system used in this study, provide richer physiological data, enabling a deeper exploration of emotional responses. Furthermore, virtual reality for remote data collection ensures a consistent and controlled environment for participants. This setting minimises external factors that could affect emotional responses and contribute to the robustness of the dataset.

**Table 1:** Physiological database summary.

Number of participants	37			
Number of videos	31 (30 affective and 1 relaxation)			
Video duration	Affective (30 s), Relaxation (120 s)			
Rating scales	Valence and Arousal			
Rating values	Discrete (1–9)			
Rating method	VR controller touch-pad			
**Number of ratings (per video)**	**Mean**	**Min**	**Max**	**Total**
Affective	24.905	0	207	27645
Relaxation	66.277	4	565	9809
**Duration (per participant)**	**Mean**	**Min**.	**Max**.	**Total**
Affective (good fit)	22m45s	14m20s	23m20s	14h2m
Total (inc. training)	27m56s	25m22s	32m25s	17h13m
Physiological Signals	EMG, PPG, IMU, Skin contact (impedance)			

The AVDOS-VR database will pave the way for extensive video validation gathering in authentic environments within and beyond the confines of research laboratories. This feat was feasible thanks to employing a self-guided protocol for data collection. Thus, given these characteristics, AVDOS-VR is a significant addition to the scarce existing affective video databases. To the best of our knowledge, it is the first publicly available VR database to show that it is possible to reliably collect physiological data remotely with limited-to-no supervision and wireless setups through participant self-guided protocol, in contrast to other database protocols^5,37^.

## Methods

### Experimental setup

**Fig. 2:**
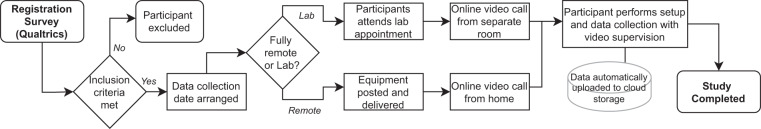
Flow diagram depicting study procedures for both recruitment variants and all segments of the study.

This study received ethical approval from the Bournemouth University Research Ethics panel (Ethics ID: 33494). Participants consented to taking part in the study and sharing their data. The study had two recruitment variants, displayed in Fig. 2. In the first variant, participants were shipped all the necessary equipment to their home addresses and the data collection was supervised via their preferred tool of video communication (Skype, Teams, Zoom etc). In the second variant, data collection was undertaken in the lab, with the supervising researcher present in an adjacent room. The supervision was provided via a video call to replicate the fully remote setting of the first variant. For this, the researcher stayed in a separate room. The reason for this second variant was mainly to speed up the data collection process by eliminating the time required for the shipment of equipment to participants.

### Recruitment and participants

Participants of the first variant of the study (fully remote data collection) were recruited via opportunity sampling from a trusted circle of friends and social affiliates because of equipment security issues. Participants were not given any incentives or reimbursement for taking part. For the second variant of the study, participants were recruited through the Bournemouth University Psychology Participant Pool System. These participants were given £20 Amazon vouchers and research credits for the successful completion of the study.

Regardless of the used recruitment method, all participants were required to complete an online registration questionnaire where their eligibility to take part was assessed. Exclusion criteria were age (below 18 or over 45 years), inability to wear contact lenses instead of glasses if eyesight correction was required, any currently diagnosed psychological conditions or any current or previous diagnoses of cardiovascular, respiratory, or neurological conditions and possible alexithymia (score: 52+) as assessed by the Toronto Alexithymia Scale (TAS-20^38^) which suggests individuals reduced ability to identify and describe experienced emotions.

Out of a total of 43 participants, 24 took part in the fully remote data collection, and 19 additional participants in the laboratory simulation of the remote data collection. Six participants were excluded in total (three from each protocol remote/lab). Of these, one participant was excluded because of possible alexithymia (score: 54) Four more participants had to be excluded due to a poor device fit. One participant was excluded because of corrupted files. The final sample consisted of N = 37 participants (21 fully remote, 16 remote lab), 16 males and 21 females. The mean age for these participants was 23.4 years (range: 18–40, SD = 5.2). None of the participants experienced motion sickness during the study, although five participants reported that they were susceptible to motion sickness. A total of 25 participants stated that they have used a VR headset at least once in the past.

### Video selection

The AVDOS-VR database, introduced in this paper, builds upon and extends the pre-existing non-VR AVDOS database. The original AVDOS database comprises 60 high-quality, emotion-evoking videos, which were previously validated through an online questionnaire^39^. Each of these videos has a precise duration of 30 seconds and is categorised into one of three emotional states: positive, neutral, or negative.

For this paper, 30 videos were selected from the existing AVDOS database for validation in VR environments using self-reported measures and physiological recordings, forming the AVDOS-VR dataset. Video IDs used throughout this study match the original AVDOS database for ease of reference and identification. Selected videos were chosen based on their original mean ratings within their respective affective categories, while also considering the videos with the smallest standard deviation (SD). This selection criteria was implemented to enhance inter-rater reliability in our study.

### Pico VR and emteqPRO systems

**Fig. 3:**
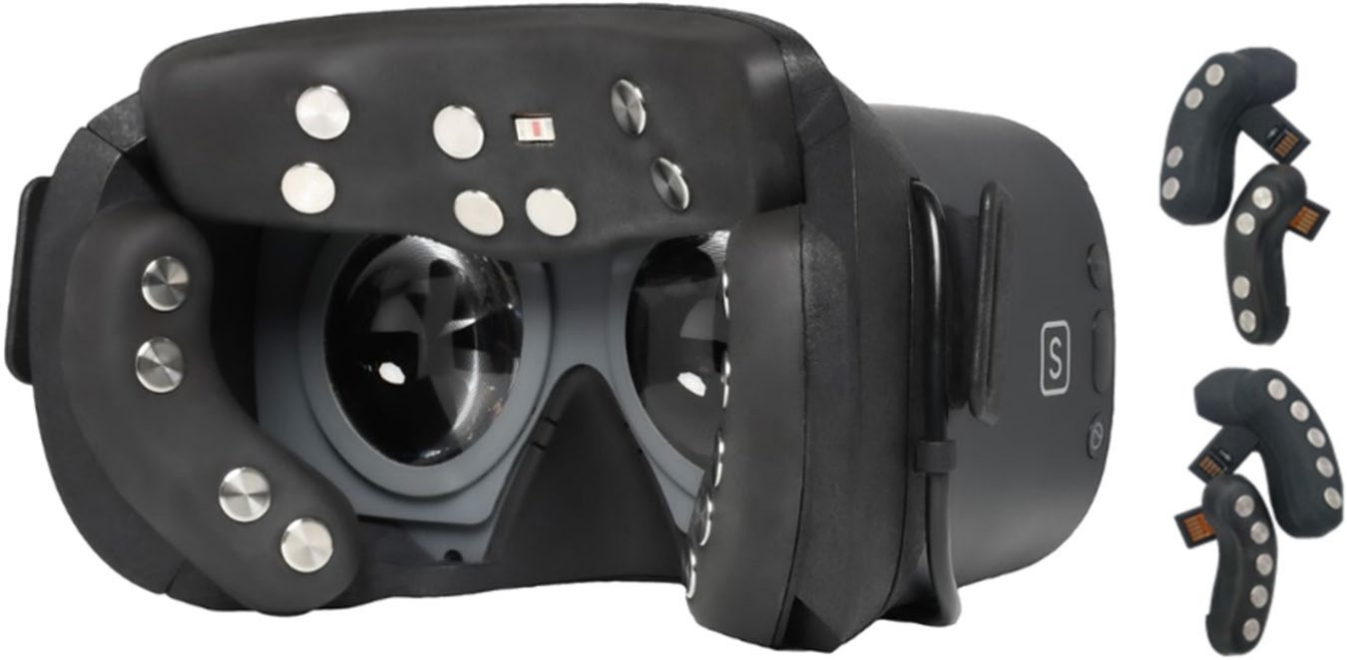
The emteqPRO Pico G2 4k model. The mask padding shows EMG electrodes and a forehead PPG sensor. Note also narrow and wide size variants of cheek sensor pairs for the emteqPRO Pico model.

Two emteqPRO/Pico devices were used for this study. The Pico G2 4k model featured a 3840 × 2160 screen resolution and a refresh rate of 75 Hz (see Fig. 3). The EmteqPro mask itself is a detachable accessory that can be mounted onto the Pico headset. For comfort, narrow and wide cheek inserts were provided to accommodate different face shapes and achieve optimal skin contact for the best signal quality. Participants could choose and replace these inserts during the initial signal check stage.

Figure 1 depicts facial muscle to EMG sensor mapping and the location of the forehead PPG sensor. The EmteqPro system produced two types of data files. The first file stored in a standard *.json* format contains custom event data pre-programmed to be triggered at specific key moments of the study like, for example, the start and end of each video. Each event has a unique timestamp which can be used to correlate events with physiological data from raw files.

**Fig. 4:**
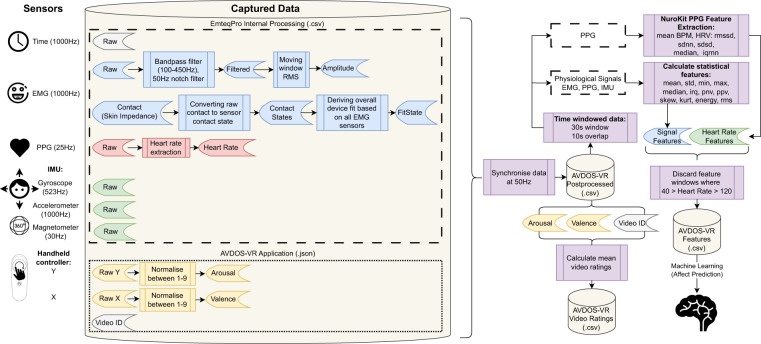
Overview of Data Processing. This diagram illustrates individual sensors, their collected data, post-processing steps, and feature extraction procedures.

The second file contained raw files *.dab* with physiological data recorded during the data collection. Namely, amplitude and contact states of facial electromyography (EMG), heart response using photoplethysmography (PPG), and movement from the inertial measurement unit (IMU). This file also contained metadata such as firmware versions, signal frequencies, and error logging. For the data analysis, the raw files were converted to *.csv* files to enhance readability using the *dab2csv* converter (dab2csv is available for download from Emteq Labs at https://support.emteqlabs.com). All the details from both raw and converted files are included in the AVDOS-VR database available online^40^. Sampling rates for each measure recorded are listed in Table 2. Raw EMG signal for the remote version of the study was recorded at 50 Hz, and the in-lab variation of the study was recorded at 1 kHz due to firmware updates made to the sensor, but the filtered EMG signal and other EMG features did not change (see^22^ for a detailed description of filtering and data processing by the emteqPRO system). The illustration in Fig. 4 provides an overview of the data processing activities conducted by both the internal emteqPRO software, custom AVDOS-VR Unity application and post-processing feature extraction in Python.

**Table 2:** List of raw recorded physiological signals.

Data type		Channels	Frequency	Description
Facial EMG*	Frame#	1		Row index for human readable data references.
	Time	1	1 kHz	Relative time in seconds at which data was measured in the hardware.
	Face State	1	—	Indicates when the device is detected as worn by the user. 0: No face contact; 1: Face contact.
	Fit State	1	—	Continuous measurement (range 0–11), where higher values represent better mask fit.
	Contact States	7	25 Hz	8-bit value denoting the contact information for each pair of EMG electrodes.
	Contact	7	25 Hz	Impedance measurement of the electrode-to-skin contact.
	Raw	7	50/1000 Hz	Raw analog signal from the EMG measurement device without filtering stages.
	RawLift	7	50 Hz	Supplementary data to internally calculate Contact States and Contact.
	Filtered	7	1 kHz	Filtered EMG measurements in the frequency ranges 100–450 Hz.
	Amplitude	7	50 Hz	Amplitude of the muscle EMG.
	Heart Rate	1	1 Hz	Average beats-per-minute (BPM) measured from the sensor on the user’s forehead.
	PPG	2	25 Hz	Raw photoplethysmography from the user’s forehead, and proximity to the sensor.
	Accelerometer	3	1 kHz	Linear acceleration for the X, Y, and Z axes.
	Magnetometer	3	30 Hz	Magnetic field strength on the X, Y, and Z axes.
	Gyroscope	3	523 Hz	Angular velocity on the X, Y, and Z axes.

*Each of the data types for Facial EMG contains the 7 channels corresponding to facial muscles:

*RightFrontalis, RightZygomaticus, RightOrbicularis, CenterCorrugator, LeftOrbicularis, LeftZygomaticus, LeftFrontalis*.

### Continuous arousal and valence ratings

Annotations for arousal and valence self-ratings were recorded using a VR controller. *x* and *y* coordinates of the finger position used for rating were normalised in the range 1 to 9. Raw finger positions on the VR controller were also recorded in the range 0 to 1.

The annotations from the circular touchpad in the VR controller were transformed to map the 2D representation of valence and arousal. This transformation was performed with a stretching method^41^ that allows corrected visual representation of the affective self-ratings using the following formula where *u*(*t*) and *v*(*t*) are the Euclidean coordinates for the emteqPRO touchpad area at time *t*:

$$x=\left\{\begin{array}{lc}{\rm{sgn}}(u)\sqrt{{u}^{2}+{v}^{2}} & :{u}^{2}\ge {v}^{2}\\ {\rm{sgn}}(v)\frac{u}{v}\sqrt{{u}^{2}+{v}^{2}} & :{u}^{2} < {v}^{2}\end{array}\right.$$

$$y=\left\{\begin{array}{cc}{\rm{sgn}}(u)\frac{v}{u}\sqrt{{u}^{2}+{v}^{2}} & :{u}^{2}\ge {v}^{2}\\ \mathrm{sgn}(v)\sqrt{{u}^{2}+{v}^{2}} & :{u}^{2} < {v}^{2}\end{array}\right.$$

The recording of a new self-rating event was triggered when a significant change in the rating was found, defined as a difference in the discrete scale for arousal and valence between 1 and 9. Small finger movements that did not result in this change were not recorded. The frequency of these self-rating changes was lower than the sampling rates for physiological measures.

### Initial setup and procedure

The entire data collection was designed to be carried out with very limited oversight or supervision (in both variants). A custom Unity application was developed to deliver the experiment and to collect the data. To this end, we built an Android application package (*.apk*) and installed it on the Android operating system running on the emteqPRO Pico model. Instructions and training sessions were integrated into the application.

In the first variant (fully remote data collection), participants were asked to charge the device before data collection, switch it on and connect a controller using on-screen instructions. Participants also connected the device to their home wifi network to enable the streaming of data to cloud storage. In comparison, in the second variant (laboratory simulation of the first variant), the device was already switched on, fully charged, and connected to the wifi network, and placed on the table in front of the participant. From that point onwards, the protocol was identical for both variants. Participants were responsible for putting the device on ensuring correct fit and comfort and launching the application. If any issues occurred, the subject and researcher would solve them via the previously established video call link.

After launching the custom application in the VR headset, participants were presented with a welcome screen. Animated instructions were utilised to teach participants how to interact with the study. A short signal quality check was performed to assess the fit quality of the device by checking an EMG sensor display. Participants were only instructed to proceed when the mask was fitted well and when the signal quality for these sensors was sufficient^22^.

**Fig. 5:**
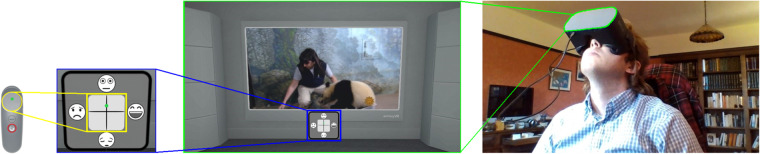
The experimental setup. From left to right we see (i) a controller used for interacting with the study, including a touch-pad used for self-ratings, (ii) the arousal and valence scale tracking finger position used for rating always displayed under the video, (iii) the video environment and (iv) a participant wearing an emteqPRO device and looking around before the experiment.

Then, subjects received an introduction to arousal and valence concepts through interactive tutorials, animations, and two training videos. They were asked next to provide continuous ratings by moving their fingers across the touch sensor area on the controller. If no finger was detected to be touching the controller, a message was displayed at the bottom of the video screen asking participants to place their finger back on the controller and resume rating. Participants had an opportunity to repeat the training session as many times as they liked until they felt comfortable with the self-rating mechanism. To help participants keep track of their ratings in real-time, an affect diagram was displayed at the bottom of the screen. This diagram utilises facial representations used in the affective slider^42^ to represent valence and arousal states (Fig. 5).

Finally, during the main video validation task, positive, neutral and negative video conditions were displayed in separate blocks. For this, ten affective videos from the same affective condition were combined into five-minute-long blocks. The order of blocks and videos within each block was randomised for each participant. A two-minute relaxation video was played before each block. This video displayed a beach scene and was reused in each block. Participants were tasked with watching and continuously rating all videos. The VR environment depicted a room with a couch and a large screen.

## Data Records

The AVDOS-VR database presented in this paper contains both raw and processed data. Data can be accessed via^43^ and the Python library used for data processing and transformation is available separately as part of a GitHub repository^44^.

### Data

Available in both compressed and uncompressed formats, ‘data’ and ‘data.zip’ directories contain raw physiological and event data. Files for individual participants can be found within data folders and are labelled in the format ‘participant_xxx’ indicating the participant number. Participants who took part in the second version of the data collection (remote lab-based) have a ‘v2’ flag at the end of the folder name ‘participant_xxx_v2’. Within each participant folder, five sets of *.csv*, *.json* and *.raw* files can be found. ‘video_1’ files include data from the training session where participants were getting familiar with the rating system. video_2, video_3 and video_4 include relaxation (shown before affective videos) and condition data for each video category (positive, negative and neutral in random order with the order of each video within the category also randomised). Finally, video_5 contains data from the last relaxation segment at the very end of the study.

- *.raw* - Raw physiological data format files. Must be converted via the 'Dab2CSV' converter included in the DabTools package provided by Emteqlabs^45^.
- .*csv* - Raw physiological data converted into comma-separated values. Refer to Table 2 for column descriptions.
- *.json* - Event data file containing timestamps and custom event labels including affective self-ratings for synchronisation between physiological signals. (See Table 3).

**Table 3:** Names and descriptions of events stored in JSON files.

Event	Description
Start of signal check	The start of signal check and data recording.
Signal check finished. Fit state: “FitState value = x”	End of the signal check. FitState, i.e., “VeryGood value = 9”
Cinema scene started	Loaded cinema scene following successful signal check
Finger lifted	Finger lifted during video segments
Finger back on touchpad	Finger placed back on the controller during video segments
Video rating training finished	End of the video training session
Category sequence: “Category_1, Category_2, Category_3”	Order of randomly selected video category sequence, i.e., “Category sequence: Positive, Negative, Neutral”
Category sequence array numbers: “x, y, z”	Numerical values of randomly selected video category sequence, i.e., “1, 2, 3”
Playing rest video	Start of the rest video played between categories
Finished playing the rest video	End of the rest video performed between categories
Playing category number: x Category name: “Category name”	Name and numerical value of the category of videos, i.e., “Playing category number: 3 Category name: Positive”
Playing video number: “x”	ID of the video being played
Finished playing video number: “x”	ID of the video which has just finished playing
Video category finished	10 videos from a video category finished playing
Valence: x, Arousal: y, RawX: x, RawY: y	Valence and arousal values. Normalised 1–9 and raw position values
Finished playing all videos	All videos have finished playing
Video ratings study: finished data recording	Video segment completed

### Python library

The Python library provided contains the code used for processing the data. Juypter notebooks were created to break down the process into a readable step-by-step process. The following notebooks are available:

- ‘0_ verify_ and_ summarize’ - verifies data completeness and generates a summary (number of ratings, time spent etc.).
- ‘1_ process_ data’ - data normalisation, feature extraction and general processing.
- ‘2_ statistical_analysis’ - presents the statistical analysis and data exploration from the features including plots.
- ‘3_ml_classification’ - produces the results running the cross-validation and hyperparameter optimisation for subject-dependent experiments.

### Processed data

The ‘Dataset_ AVDOSVR_ postprocessed.csv’ file contains 50 Hz, normalised, filtered and labelled data from all participants used for feature extraction and consecutive steps. This file is a result of the ‘1_ preprocess_ and_plot’ notebook. Lastly, the file ‘video_ratings.csv’ contains mean valence/arousal values, and mean and total number of ratings per video.

## Technical Validation

### Study protocol and data quality

EmteqPRO devices offer a real-time fit assessment metric for individual EMG sensors (Emg/ContactStates, see Table 2 or the device manual^40^). These EMG sensors can have various states, including “lifted” (no skin contact), “contact” (initial or intermittent skin contact), “stable” (firmly established contact), “fault” (indicating a faulty contact), and “settled” (stable with saturated filters, indicating higher measurement confidence in Emg/Filtered and Emg/Amplitude).

The overall device fit is estimated based on the EMG/Contact states taking all sensors into account, resulting in fit values ranging from −1 to 11. These values offer a straightforward device fit metric, where −1 indicates fit detection failure, 0 signifies the device is not on the user’s face, and 11 represents ideal sensor impedance (although not achievable on the user’s face). Higher values indicate a better device fit.

To initiate data collection, participants were required to adjust the device until the average fit reached a recommended threshold (8, denoting general functionality, i.e., all sensors making contact with the skin with at least 5 out of 7 pairs reaching settled status), as per the manufacturer’s manual. The current fit value was displayed to participants during the initial device calibration, and they were instructed to continue adjusting the device until this threshold was met, at which point data collection began. However, it is important to note that fit quality might degrade over time or immediately after calibration ends.

**Fig. 6:**
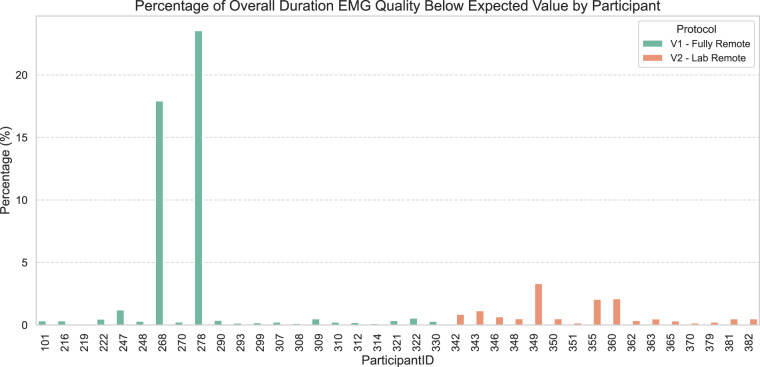
Signal quality comparison between remote and in-lab recordings as measured in percentage of overall time where the device was worn below the expected level of fit. The figure shows all participants and a low amount of undesirable fit for both study protocols.

To evaluate signal quality for each participant across the two protocols (fully remote and remote lab), we calculated the duration in seconds during which the fit quality fell below the desired threshold. Figure 6 provides a visual representation of the time spent (percentage of overall duration) below the target average fit for each participant.

The device fit was below the desired threshold for 600.14 seconds in the remote version of the protocol and 173.12 seconds in the lab version. Notably, out of the 600.14 seconds in the remote version, 522.48 seconds were attributed to just two participants. Despite this imbalance, the Mann-Whitney-U test revealed no significant difference between the two protocols, although the p-value approached significance (U = 231, p = 0.055).

The time of poor contact for these two participants (225.86 and 296.62 seconds individually) constitutes a relatively small portion of the overall study duration (mean of 27 minutes and 56 seconds, or 1676 seconds). Nevertheless, the nearly significant results could suggest that remote data collection warrants additional scrutiny, with lab data being marginally superior. However, when we treat these two participants as outliers and exclude them from the analysis, the Mann-Whitney test becomes more significant (U = 231, p = 0.009) with 77.66 seconds of poor contact in the fully remote vs 173.12 in the lab version, suggesting the opposite result of remote data being more reliable.

In summary, this analysis indicates that neither of the two protocols provided significantly superior data quality. Unsupervised and fully remote data collection carries the potential for more fundamental issues, such as participants moving or removing the device during the study, which a supervising researcher would quickly notice. Both supervised and unsupervised remote data may have a higher likelihood of erroneous data bypassing initial checks, necessitating more rigorous validation procedures.

### Self-reported affect ratings

**Fig. 7:**
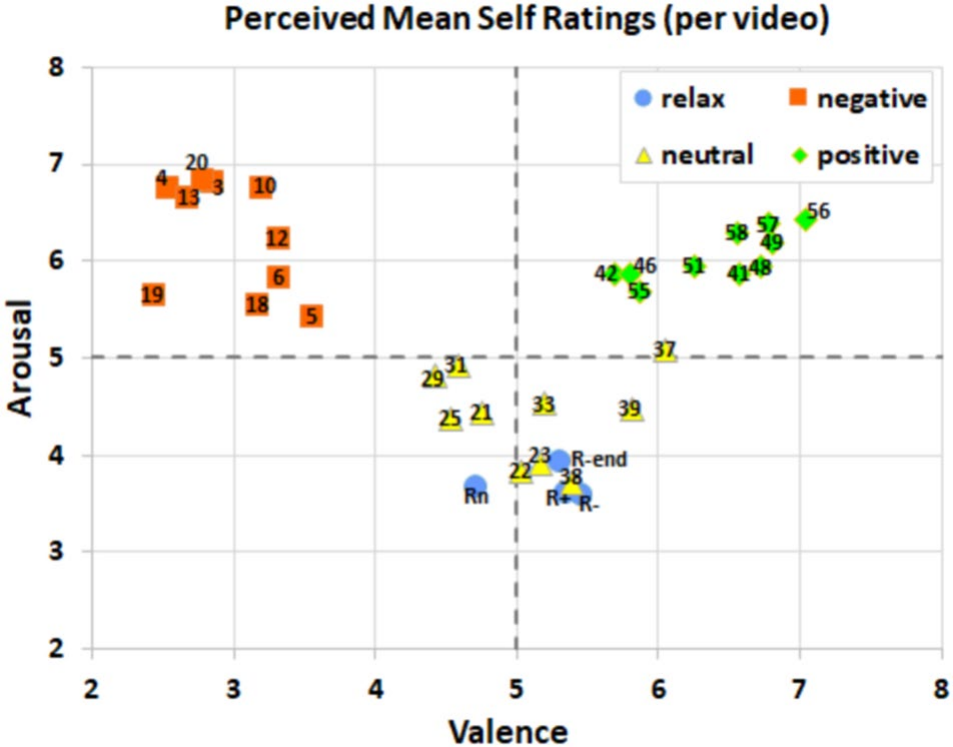
Valence and arousal self-ratings for each video averaged across participants and grouped per video condition (positive, neutral, negative). Blue circles represent ratings of the resting video that was played before each block and at the end of the task.

Self-ratings/annotations of arousal and valence were recorded continuously for all videos. Ratings from all participants were aggregated for each video to compute average valence and arousal ratings. Results are shown in Fig. 7. We validated whether the average self-reported ratings in valence and arousal differed between the three affect-type conditions. Continuous self-ratings were grouped by participant to calculate mean arousal and valence ratings per block for each participant (N = 37). We used Shapiro-Wilk tests to check for normality in valence ratings. Negative and neutral valence ratings were normally distributed ($W(36)=0.970,p=0.411$ and $W(36)=0.973,p=0.487$ respectively), while positive valence ratings were not $W(36)=0.773,p < 0.001$. Friedman testing showed that the mean reported valence was significantly different in all three conditions (${\chi }^{2}(2)=66.378,p < 0.001$, post-hoc Wilcoxon signed-rank tests for neutral vs negative, $W=703,p < 0.001$; positive vs neutral, $W=665,p < 0.001$; positive vs negative conditions, $W=698,p < 0.001$).

**Fig. 8:**
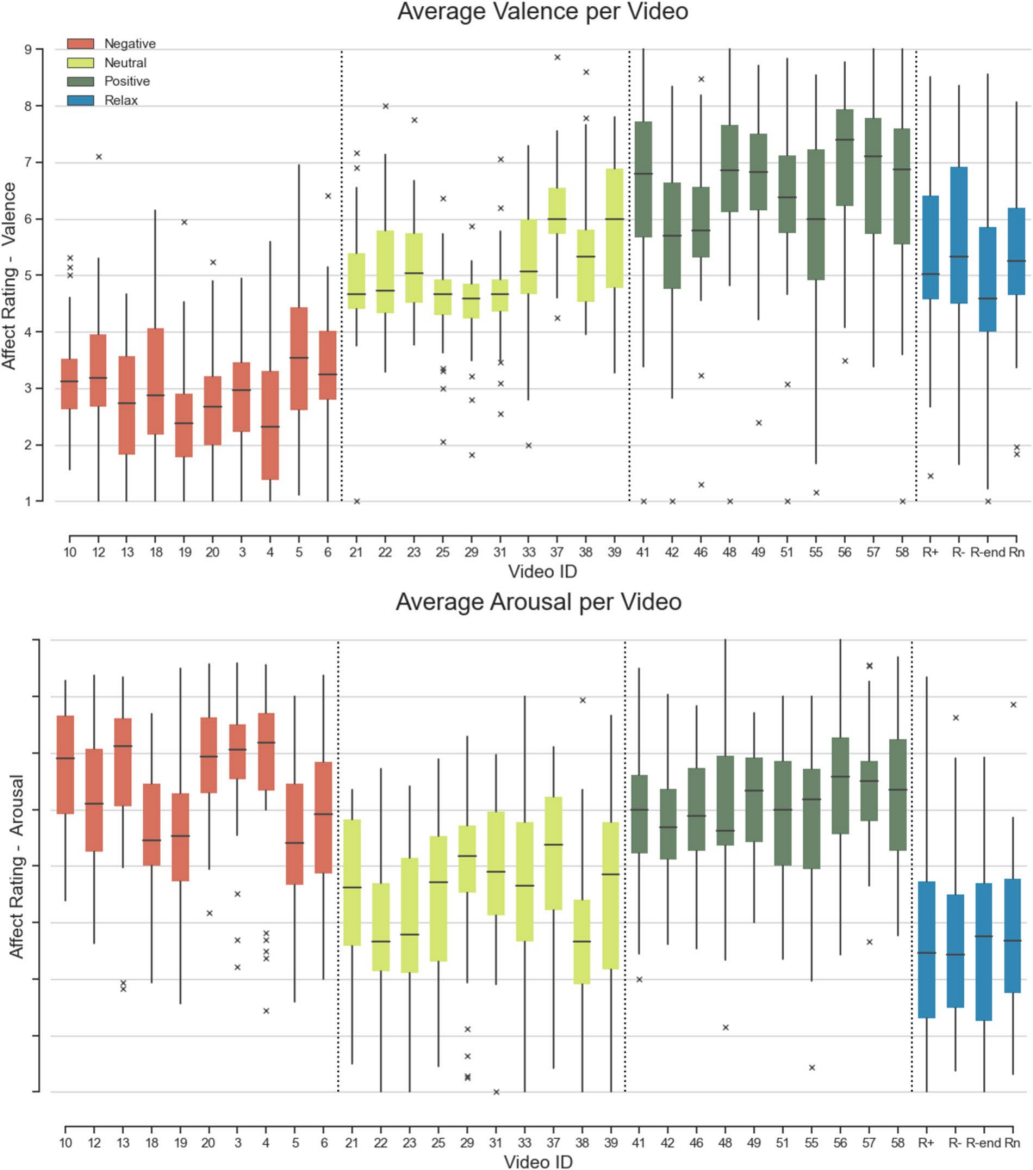
Valence and arousal self-ratings (1-9) for each video, averaged across participants and grouped per video condition (positive, neutral, negative). R + (positive), R- (negative) and Rn(neutral) are segments where the relaxation video is played before each corresponding category. R-end was the same relaxation video played at the end of the study.

**Fig. 9:**
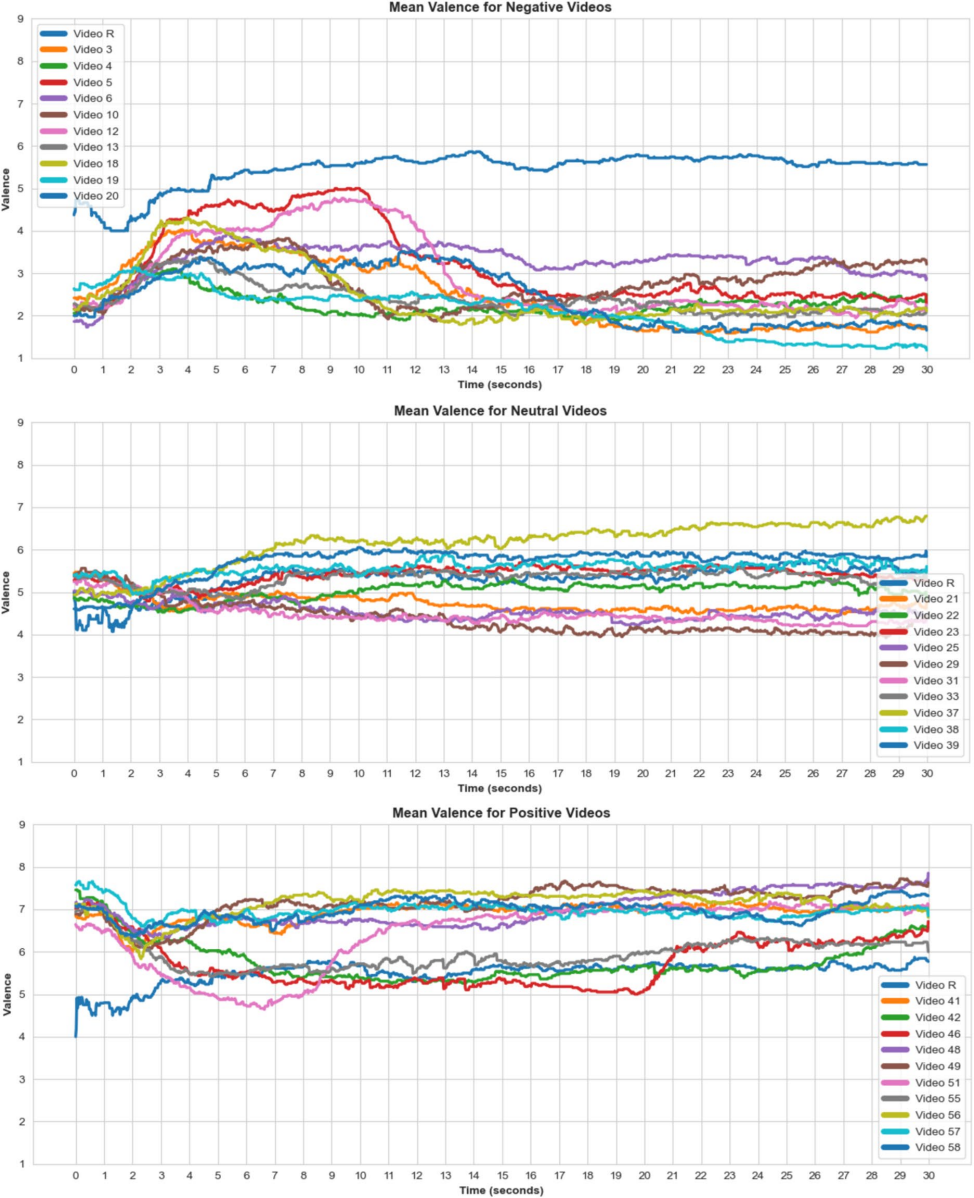
Valence ratings for the entire video duration, means across all participants. Video R is the relaxation video. Sudden change in affective ratings in some videos can be a result of a sudden event within a video such as a whale unexpectedly breaching the water surface.

**Fig. 10:**
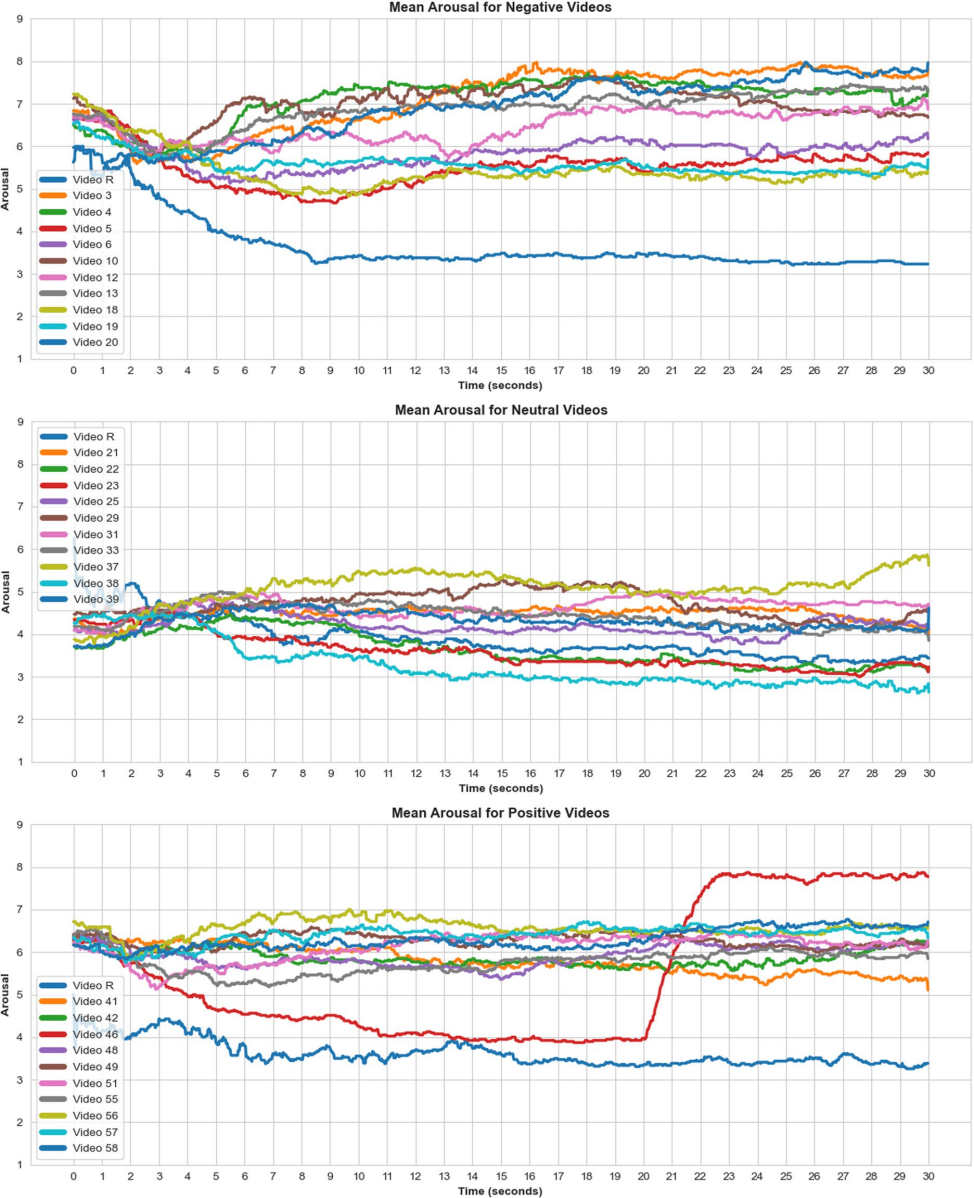
Arousal ratings for the entire video duration, means across all participants. Video R is the relaxation video. Sudden change in affective ratings in some videos can be a result of a sudden event within a video such as a whale unexpectedly breaching the water surface.

We applied an identical approach to arousal ratings. Shapiro-Wilk tests showed only positive arousal ratings were normally distributed ($W(36)=0.975,p=0.556$), while negative ($W(36)=0.940,p=0.045$) and neutral ($W(36)=0.940,p=0.047$) arousal ratings were not. Friedman test showed arousal ratings were likewise significantly different for all three conditions (${\chi }^{2}(2)=42.378,p < 0.001$). Post-hoc Wilcoxon signed-rank showed arousal ratings for both negative and positive conditions were significantly higher compared to the ratings in the neutral condition ($W=693,p < 0.001$; $W=694,p < 0.001$ respectively). As expected, there was no significant difference in arousal for negative and positive conditions ($W=210,p=0.984$). The boxplot in Fig. 8 depicts average valence and arousal ratings for each video block across all participants. In addition, Figs. 9, 10 show changes in valence and arousal ratings over time for each video.

### Physiological measures

Physiological signals were processed to study the variation of measures for each of the three experimental conditions (positive, neutral and negative). First, a subset of the available signal features was selected (see Table 2). Namely, the EMG amplitude and contact data for each of the seven facial EMG channels, PPG sensor data to calculate mean heart rate (HR) and heart rate variability (HRV) measures, and IMU sensor data to analyse motion-related measures corresponding to accelerometer, magnetometer, and gyroscope.

We first divided the time series of the selected raw physiological signals into analysis segments using event markers identifying each of the experimental conditions. These segments were then either down-sampled (accelerometer, gyroscope) or linearly interpolated (EMG contact, PPG and magnetometer) to match the facial EMG amplitude sampling frequency of 50 Hz. Data samples were removed if the corresponding faceplate’s fit state was lower than 8, which is the threshold indicating an average abstract measure of mask fit with all EMG sensors making skin contact (minimum reliable value recommended for this device^45^).

The annotations with the continuous affective self-ratings have irregular sampling frequencies, therefore, they were merged with their corresponding physiological data using forward filling (propagating the last known reported rating until a new self-reported value is recorded).

Next, data were normalised (*μ* = 0, *σ* = 1) for each participant individually by using physiological data from all segments. Then, features were extracted using sliding windows with 30 s width and 10 s overlap. The 30-second window of 1500 initial patterns (at a resampled frequency is 50 Hz) is discarded if this number drops below 95% due to filtering (thus, the smallest time window consists of 1425 data points).

#### Feature extraction

All variables were processed with the following statistical features: Mean, Standard Deviation (Std), Minimum Value (Min), Maximum Value (Max), Median, Interquartile range (IRQ), the proportion of negative (PNV) and positive (PPV) values, skewness, kurtosis, energy, and RMS.

#### Heart rate and heart rate variability analysis

**Fig. 11:**
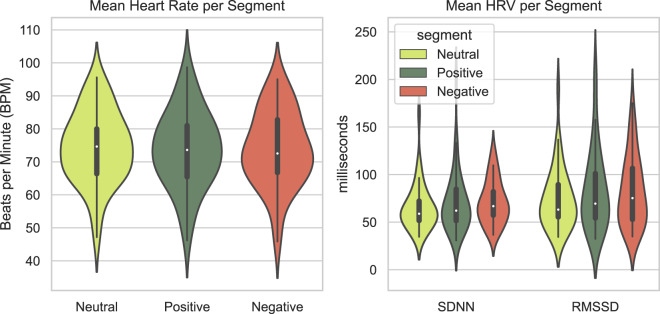
Violin plots depicting mean heart rate (BPM) and HRV (milliseconds) for the positive, neutral, and negative conditions.

In addition to these statistics, the PPG signal enables us to extract mean heart rate (HR) in beats per minute (BPM) and heart-rate variability (HRV) features, including standard deviation of the RR intervals (SDNN) and square root of the mean of the squared successive differences between adjacent RR intervals (RMSSD). We filtered outliers for any 30s-long window with HR outside the interval 40 to 120 BPM. Afterwards, the raw PPG signal was processed using the NeuroKit Python library^46^. The mean heart rate and HRV were calculated for each condition separately, providing one mean HR value for each on the neutral, positive and negative conditions. They are displayed in Fig. 11. No significant differences between mean heart rates were found between conditions (One-way ANOVA, $F(2,34)=0.04,p=0.961$). Likewise, HRV did not differ between the three conditions both for the SDNN measure ($F(2,34)=0.507,p=0.603$) and for the RMSSD measure ($F(2,34)=0.618,p=0.541$).

#### Facial EMG analysis

**Fig. 12:**
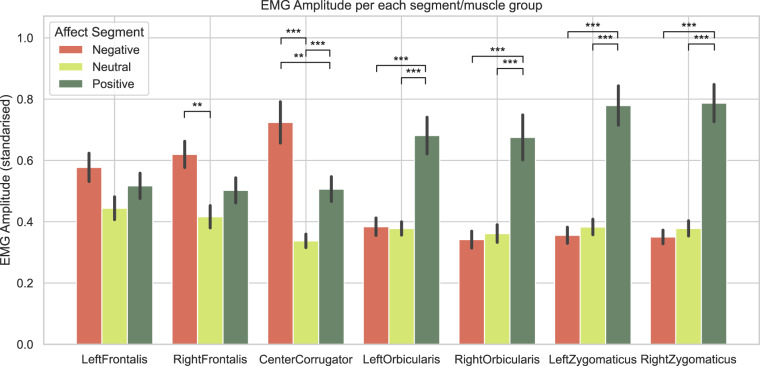
RMS EMG Amplitude for each segment. A participant-specific standardisation was applied. Post-hoc paired t-tests results: **p < 0.01, ***p < 0.001.

Facial mean EMG responses were calculated by averaging Emg/Filtered signal RMS (root mean square) over moving time windows separately for each condition and facial muscle group. Figure 12 displays between-conditions comparisons for each muscle group. As expected, positive videos rendered the highest activation in the zygomaticus (smile) and orbicularis (eye) muscles. By contrast, negative videos showed the highest activation of the corrugator (frown) muscle while also activating orbicularis muscles but to a lesser extent than positive videos. One-way ANOVA tests showed highly significant differences between conditions for all muscle groups (p < 0.001) except left frontalis (*F*(2, 34) = 0.81, *p* = 0.12). Post-hoc paired t-tests (Bonferroni-corrected) were used to analyse differences between the specific conditions for each muscle group. These results are also displayed in Fig. 12.

#### Motion analysis

For the motion analysis, positive, neutral and negative conditions were compared for the z-axis (backward and forward movements), separately for the accelerometer, magnetometer and gyroscope motion data. The z-axis was chosen because the approach-avoidance hypothesis suggests that both negative and positive categories should contain more backward and forward movement than the neutral category.

**Fig. 13:**
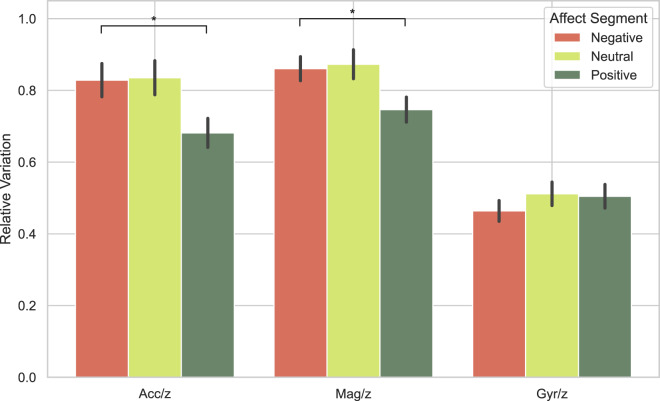
Variation of motion data recorded on the Z-axis (backwards and forwards) as measured by the accelerometer, magnetometer and gyroscope sensors and displayed for the positive, neutral and negative conditions separately.

Magnetometer and acceleration sensors registered more movement on the z-axis (backwards and forwards) in negative and neutral categories than in positive (Fig. 13). Acceleration data showed significant differences between conditions (One-way ANOVA, ($F(2,34)=3.753,p=0.027$), as did magnetometer data ($F(2,34)=3.677,p=0.029$), while gyroscope data was not significantly different ($F(2,34)=0.668,p=0.515$). Post-hoc t-tests for the acceleration data showed differences in the amount of z-axis for the positive vs negative conditions ($t(36)=2.531,p=0.008$) while failing to distinguish between the other two conditions (positive vs neutral: $t(36)=2.284,p=0.986$; negative vs neutral: $t(36)=0.080,p=0.532$). For the magnetometer data, similarly to acceleration, post-hoc t-tests showed a difference between positive and negative conditions $t(36)=2.401,p=0.011$), but no differences between the other two conditions (positive vs neutral: $t(36)=2.301,p=0.986$; negative vs neutral $t(36)=0.200,p=0.579$).

### Feasibility of affect classification with physiological AVDOS-VR measures

#### Experimental setup

This section provides a simple example of the effective usage of this new dataset for affect classification. This proof-of-concept method consists of classifying three levels of valence (negative, neutral, positive) and two levels of arousal (high and low). The ground-truth labels were defined as the video condition of the data set. The statistical validation of self-ratings suggests that video conditions are good indicators of the perceived valence and arousal levels and, hence, that they can be reliably used as class labels.

##### Preprocessing

Physiological signals were resampled, merged and processed as described: Features were extracted from 37 subjects to generate a data frame containing 2329 observations and 320 columns with physiological features and their respective class labels. The classification task was performed with all 37 participants after applying the exclusion and filtering. The processed dataset is freely available in the project’s repository.

##### Data modalities

The 318 physiological features (described in subsection *Feature extraction*) were grouped in data modalities for the classification task. In total, 42 features corresponded to HRV, 108 to IMU data, 84 for the EMG amplitude (EMG-A), and 84 for the EMG contact impedance values (EMG-C). The annotations from the continuous self-reported arousal and valence were processed to extract 12 statistical features used as the inputs for determining the *baseline* classification results (Table 4, see arousal and valence classification in the results section).

**Table 4:** Average F1-scores over 37 participants, grouped by affective target, classifier, and data modality.

Target	Arousal (2-classes)					Valence (3-classes)				
Classifier	DL	SVM	KNN	Linear	RF	DL	SVM	KNN	Linear	RF
**Annot**.	0.77	**0.84**	0.82	0.82	0.81	**1.00**	0.79	0.77	0.77	0.79
**EMG A**	0.77	0.76	0.73	0.75	**0.78**	0.75	**0.79**	0.73	**0.79**	0.78
**EMG C**	0.54	**0.56**	**0.56**	0.52	0.55	**0.60**	0.40	0.39	0.39	0.38
**HRV**	0.51	0.48	0.54	0.54	**0.55**	0.27	0.33	**0.36**	0.34	0.33
**IMU**	0.61	0.64	0.56	**0.65**	0.61	**0.57**	0.50	0.39	0.47	0.41
**Mask-All**	0.74	0.73	0.67	0.73	**0.78**	0.73	0.75	0.63	0.75	**0.77**

Bold values signify the best classifier performance for each modality. Annotation refers to continuous self-reported rating and Mask-All refers to the combination of all modalities from the acquisition device. Random baseline classification accuracy is 0.5 for arousal and 0.33 for valence.

##### Classifiers

As an example of the database capabilities, each data modality was employed to train four traditional machine learning classifiers commonly used for affect recognition^47^, and one deep learning (DL) model^48^. The classifiers comprised a ridge linear regression (where the output was categorised for classification) endowed with Tikonov regularisation optimised within the range $\gamma \in [1{0}^{-5},1{0}^{5}]$, an SVM with a Gaussian kernel optimised for the variance $\sigma \in [1{0}^{-1},1{0}^{-3}]$ and optimal regularisation $c\in [1,1{0}^{3}]$, a random forest optimised in the number of trees $N\in \{10,50,100\}$ and depth $D\in \{5,10,20\}$; and a K-nearest neighbours classifier with $n\in \{1,5,11,15\}$. The DL model was implemented as a shallow neural network using the Scikeras wrapper to integrate with the evaluation pipeline implemented in Scikit-learn. Networks used categorical cross-entropy loss function and an Adam optimiser with learning rate $\alpha \in [0.05,0.001]$ and dropout rate $p\in [0,0.05]$. A shallow architecture (one hidden layer) and a two-hidden layers network were implemented with 100 and 50/50 units respectively. This architectural structure has been chosen because deeper convolutional networks for affect recognition (see recent reviews in^49,50^) can be prone to over-fitting for EMG inputs, especially in datasets of comparable sizes to the AVDOS-VR EMG database^50^.

##### Evaluation

Each combination of data modality and classifier was evaluated with *nested* leave-one-subject-out cross-validation (LOSO-CV), a standard two-stage approach for hyperparameter optimisation, followed by a robust, subject-independent out-of-sample validation with fixed hyperparameters. Each participant was treated as a test subject once, while the remaining data was used for training. This process was repeated for each participant, and the performance metrics were averaged across all iterations to obtain the final evaluation of the algorithm’s performance. The best classifier is chosen based on the out-of-sample performance, as measured by the F1-score. The analysis was implemented in Python 3.9 (using the aforementioned libraries) and conducted on an Ubuntu 18.10 machine with AMD 2950X CPU, 128GB RAM, and two GPUs NVidia RTX 2080Ti.

#### Arousal and valence classification

Optimal hyperparameters are chosen for each combination of data modality, classifier, and test subject based on the out-of-sample overall classification performance. Based on the average ratings for each video category presented in Fig. 7, arousal classification was defined as a 2-class problem combining positive and negative videos (class 1), and neutral videos (class 0). Valence classification was defined as a 3-class problem identifying each video category independently (negative: −1, neutral: 0, positive: 1).

Table 4 shows the F1-score obtained with the AVDOS-VR dataset and averaged over 37 participants. Annotations are a reliable proxy for the ground truth (the video categories), assuming that self-reported continuous ratings yield the necessary information to predict the intended affect accurately (see below). Thus, we term the decoding performance using annotations *baseline* results. Then, each physiological modality captured is used individually as input. ‘Mask-all’ results combine all physiological signals/modalities captured by the device and do not include participants’ self-rating annotations.

Baseline results confirm that self-reported ratings yield a nearly perfect score of 1.0 (0.995014, rounded up) for a 3-class valence classification with the DL model (Table 4), as expected. Consequently, Fig. 8 depicts the close mapping between video categories and participants’ self-reported valence, despite the subjective nature of self-assessments introducing some variability. Negative and Positive valence videos (red and blue box plots) feature distinctly lower and higher self-ratings; neutral stimuli provide comparable ratings to relaxation videos (green). This correlation underlies the high baseline performance of the expressive DL classifier.

By contrast, the optimal arousal classifier F1-score drops to 0.84. This high but sub-optimal performance reflects the less salient relationship between arousal self-ratings and video categories by design since videos are optimised for valence elicitation (see Methods).

When classifiers consider all physiological measures for deriving input features, their performance reaches a 0.78 F1-score for arousal and 0.77 for valence, below the baseline, as expected. The random forest performs best for arousal, and the DL model is best for valence.

**Fig. 14:**
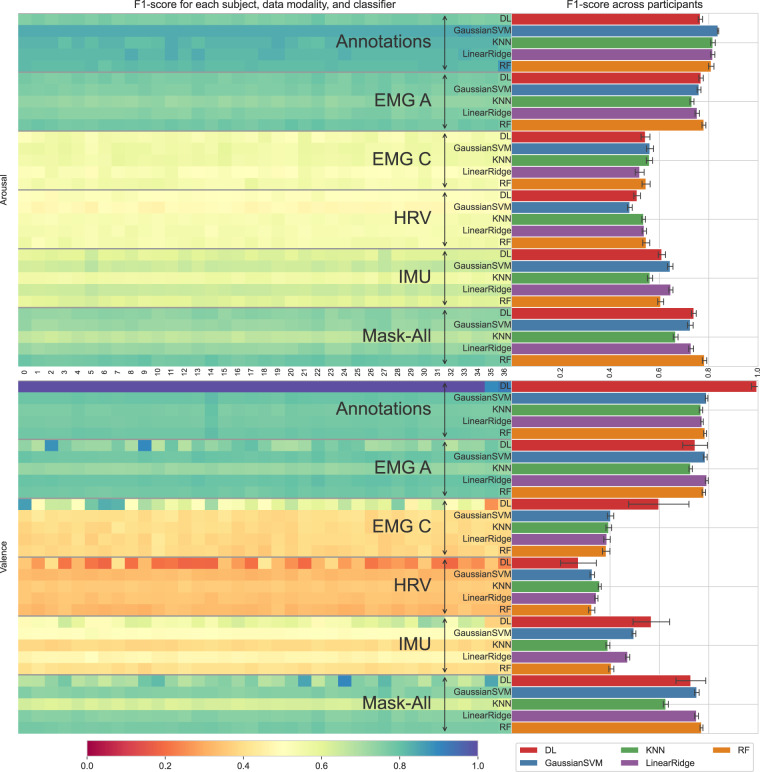
This composite figure integrates a bar plot and a heatmap to illustrate classification performance, as assessed by the F1-score, across all classifiers, modalities, and participants. The heatmap visually presents the performance of each participant, with the x-axis representing participant IDs. Additionally, the bar plot depicts the average classifier performance across all participants for each classifier and modality combination.

Classification scores for the individual data modalities show that PPG features are the least informative for arousal (0.55) and valence (0.36) classification, producing almost the same F1-scores as chance (0.5 and 0.33, respectively). The EMG amplitude is the most influential modality for affect detection, with F1-scores of 0.78 and 0.79, better than when all physiological modalities are combined. The achieved classification performance for binary arousal and 3-class valence is higher than recent datasets for VR-based affect recognition^51^. A visual comparison of the averaged F1-scores across participants is also visually presented in Fig. 14, where the error bars indicate standard deviations across the participants.

#### Subject-specific feature importance

The heatmap in Fig. 14 shows the best F1-score achieved for each subject, per combination of data modality, classifier, and target variable. The labels in the centre indicate the subject ID used as the test set and the average F1 scores across participants. The legend below refers to the F1 scores in the heatmap plot and the corresponding classifier in the barplot. Results depict that EMG amplitude is the most reliable physiological modality for both arousal and valence classification (consistent with Table 4). In addition, the LOSO-CV employed for the evaluation allows discriminating subject-dependent responses that may be directly related to the target variables^47,52^. For instance, the DL model for valence recognition produced high F1 scores in some specific participants even though their data were not included during the training stage. Namely, F1 scores higher than 0.8 were achieved only with EMG amplitude in subjects 2 and 9; only with EMG contact impedance in subjects 0, 6, and 7; or a combination of all features from the mask in Participant 24. Subject-specific responses were similar across participants for arousal classification and traditional ML classifiers in both target variables.

## Usage Notes

Researchers have the option to use processed data ‘Dataset_ AVDOSVR_ postprocessed.csv’. This includes labelled and processed data conveniently prepared and ready for feature extraction. For those wishing to develop different processing methods, raw data is also available.

### Timestamps

Event timestamps stored in *.json* files use J2000 format and must be converted to synchronise with physiological signals if not using a post-processed data file. An example timestamp from an event file is ‘676562930518’. To convert it to a common Unix timestamp format used by the raw physiological data files, a constant of 30 years of milliseconds needs to be added to our event timestamp: 676562930518 + 946684800000 = 1623247730518. The resulting Unix timestamp can then be easily decoded using numerous built-in libraries or online tools^53^. For the raw data, the Unix timestamp of the start of the recording is saved in the metadata ‘#Time/Seconds.unixOffset’. This can be used in combination with the ‘Time’ column which stores the number of milliseconds since the start of the recording to synchronise custom events and physiological data observation rows.

## Data Availability

Data processing was carried out in Python (v3.9) and all code developed for its pre-processing, transformation and analysis is user-friendly, documented, and freely available via our Github repository^44^.
